# Comprehensive In Silico Characterization of the Coding and Non-Coding SNPs in Human *Dectin-1* Gene with the Potential of High-Risk Pathogenicity Associated with Fungal Infections

**DOI:** 10.3390/diagnostics13101785

**Published:** 2023-05-18

**Authors:** Hakeemah H. Al-nakhle, Aiah M. Khateb

**Affiliations:** 1 Department of Medical Laboratory Technology, Collage of Applied Medical Science, Taibah University, Medina 42353, Saudi Arabia; hnakhly@taibahu.edu.sa; 2 Special Infectious Agents Unit, King Fahd Medical Research Center, King Abdulaziz University, Jeddah 21589, Saudi Arabia

**Keywords:** bioinformatics, *CLEC7A* gene, diagnosis, *Dectin-1* protein, fungal infection, nsSNPs

## Abstract

The human C-type lectin domain family 7 member A (*CLEC7A*) gene encodes a *Dectin-1* protein that recognizes beta-1,3-linked and beta-1,6-linked glucans, which form the cell walls of pathogenic bacteria and fungi. It plays a role in immunity against fungal infections through pathogen recognition and immune signaling. This study aimed to explore the impact of nsSNPs in the human *CLEC7A* gene through computational tools (MAPP, PhD-SNP, PolyPhen-1, PolyPhen-2, SIFT, SNAP, and PredictSNP) to identify the most deleterious and damaging nsSNPs. Further, their effect on protein stability was checked along with conservation and solvent accessibility analysis by I-Mutant 2.0, ConSurf, and Project HOPE and post-translational modification analysis using MusiteDEEP. Out of the 28 nsSNPs that were found to be deleterious, 25 nsSNPs affected protein stability. Some SNPs were finalized for structural analysis with Missense 3D. Seven nsSNPs affected protein stability. Results from this study predicted that C54R, L64P, C120G, C120S, S135C, W141R, W141S, C148G, L155P, L155V, I158M, I158T, D159G, D159R, I167T, W180R, L183F, W192R, G197E, G197V, C220S, C233Y, I240T, E242G, and Y3D were the most structurally and functionally significant nsSNPs in the human *CLEC7A* gene. No nsSNPs were found in the predicted sites for post-translational modifications. In the 5′ untranslated region, two SNPs, rs536465890 and rs527258220, showed possible miRNA target sites and DNA binding sites. The present study identified structurally and functionally significant nsSNPs in the *CLEC7A* gene. These nsSNPs may potentially be used for further evaluation as diagnostic and prognostic biomarkers.

## 1. Introduction

Human *Dectin-1* is a type II membrane protein expressed in many myeloid cells, including macrophages, monocytes, dendritic cells, neutrophils, and gamma delta T cells from the lymphoid lineage [1,2,3,4]. It plays an essential role in pathogen recognition and signaling in innate immunity [5,6]. Recently, *Dectin-1* was found to increase immunological tolerance by binding to the conserved core domain of annexins (annexins A1, A4, and A13) produced on apoptotic cells [6]. It has a cytosolic portion containing an immunoreceptor tyrosine-based activation motif termed ITAM, an extracellular lectin domain, and a transmembrane domain. It has 10 isoforms listed in the UniProt database [7]. It contains a signaling motif known as hemITAM in its intracellular cytoplasmic tail [7], and two conserved amino acids (Trp 221 and His 223) are critical for ligand-*Dectin-1* receptor interactions since they contribute to the binding sites and functions of the receptor [7]. The *CLEC7A* isoform 1 consists of 247 amino acids and serves as a pattern-recognition receptor (PRR) that recognizes β-1,3-linked and β-1,6-linked glucans, which form the cell walls of pathogenic bacteria and fungi [8]. *Dectin-1* lacks Ca^2+^-binding sites in its CRD as it does not require Ca^2+^ for ligand recognition [9]. However, β-glucan Ca^2+^ binding sites have shown that bound calcium ions (Ca^2+^) are needed to stabilize the domain structure [10]. *Dectin-1* binds specifically to β-glucan receptors, requiring at least 10- or 11-mer chain length in *Dectin-1*’s lectin domain [11]. Increasing β-glucan chain length correlates with increasing secondary structure formation, thus increasing the interaction as the helical structures fit into the ligand-binding site of the *Dectin-1* lectin domain [12]. *Dectin-1* only recognizes the middle part of the β-glucan chain, not the reducing/non-reducing ends [12]. In fungal recognition, on macrophages and DCs, *Dectin-1* accomplishes two tasks: it internalizes β-glucan-containing particles and sends signals into the nucleus [12].

Single point mutations (SNPs), including synonymous (sSNPs) and non-synonymous (nsSNP) SNPs, have been shown to contribute to the pathophysiology of fungal infection. In a family with mucocutaneous fungal infections, a recessive mutation that changed an amino acid in *Dectin-1* (Y238X) was discovered. Leading to the prematurely terminated translation of the *Dectin-1* receptor’s tyrosine residue [13]. Even if fungal killing and phagocytosis happen regularly, this mutation has been linked to susceptibility to fungal infections [14]. The final 9 amino acids of the CRD are deleted due to a nonsense mutation brought about by a single nucleotide alteration in this gene. Upon fungal infection or challenge with β-glucan, the mutation exhibits the expected loss of function and decreased cytokine response—changes in the translation termination at the tyrosine residue of the *Dectin-1* receptor. The mutations in *CLEC7A* (SNPs rs3901533, rs7309123, and rs16910527) have been associated with fungal infection susceptibility, and *Dectin-1* genetic variation plays a crucial role in fungal infection. Meta-analysis results suggest that *CLEC7A* SNPs may affect profound fungal infection susceptibility. In contrast, polymorphisms in rs16910526 are unlikely to have a significant effect.

Further investigations are warranted to verify and extend the present results and design novel immunotherapeutic strategies to optimize or replace conventional antifungal treatments. Previous studies used nsSNPs as computational tools to understand the molecular mechanisms of various diseases [15]. These tools are crucial in developing personalized medicine based on genomic variation and structural and functional analysis of nsSNPs. The impact of nsSNPs on the *Dectin-1* protein on fungal disease pathogenesis was not fully understood. This study aimed to identify potential coding and non-coding SNPs that may affect *Dectin-1* protein function, utilizing various computational approaches and bioinformatics tools.

## 2. Materials and Methods

### 2.1. Retrieval of Dectin-1 nsSNPs

**Figure 1:**
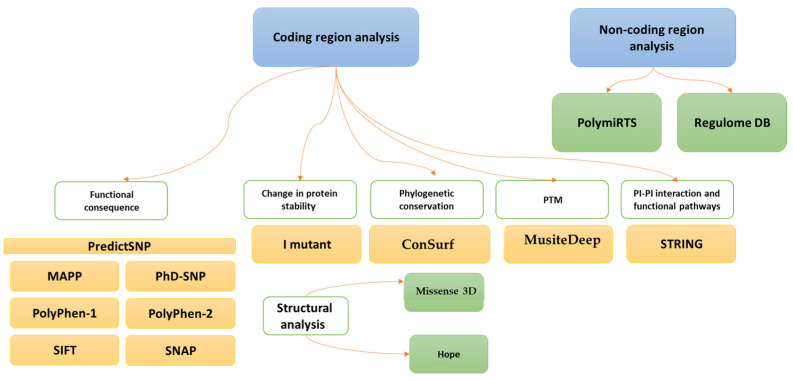
An overview of in silico analysis used in this study.

SNPs of the *Dectin-1* gene were retrieved from the dbSNP database (https://www.ncbi.nlm.nih.gov/snp (accessed on 7 April 2023)). Primarily, 975,620 SNPs were found, but after applying the missense, pathogenic, and frequency filters, 2068 SNPs were retrieved. The 247 nsSNPs in the coding region were selected for further analysis as the change in codon results in different amino acids. To analyze the non-coding region SNPs, the ENSEMBL database was used to collect the dataset for the *Dectin-1* protein. An overview of the methodological approaches is summarized in Figure 1.

### 2.2. Prediction of Functional Effects of Pathogenicity of nsSNPs

PredictSNP, which combines six software tools, was used to precisely analyze and predict the pathogenicity of *Dectin-1* nsSNPs and find the highly risky and deleterious SNPs that can significantly alter the structure or function of *Dectin-1* protein. The software includes MAPP, PhD-SNP, PolyPhen-1, PolyPhen-2, SIFT, and SNAP. As a result of combining the six best-performing tools into a consensus classifier, PredictSNP, prediction performance improved significantly. At the same time, all mutation results were returned, confirming that consensus prediction offers a more reliable and accurate alternative to individual tool predictions [16].

### 2.3. Determining nsSNPs on the Domains of Dectin-1

InterPro software was used to locate the site of nsSNPs on the conserved domains of *Dectin-1* [17] (https://www.ebi.ac.uk/interpro/ (accessed on 7 April 2023)), which can identify motifs and domains of a protein. Consequently, the software was able to determine the functional characterization of a protein using the database consisting of protein families, domains, and functional sites [18].

### 2.4. Analyzing Protein Evolutionary Conservation

The ConSurf server identifies the evolutionary conservation of the amino acids in the protein sequence and analyzes the phylogenetic relationships between homologous sequences [19,20,21]. The conserved nsSNPs of *Dectin-1* were considered for further analysis.

### 2.5. Analyzing the Effect of the nsSNPs on Protein Stability

To identify the effect of the damaging nsSNPs on the structure and stability of the *Dectin-1* protein, I-Mutant 2.0 was used [22] (https://folding.biofold.org/i-mutant/i-mutant2.0.html (accessed on 7 April 2023)). I-Mutant 2.0 is another SVM-based tool that measures the change in free energy (Delta Delta G) and predicts whether it is increasing or decreasing. A Delta Delta G (DDG) (kcal/mole) value of 0 indicates a decrease in protein stability, whereas a DDG (kcal/mole) value > 0 indicates an increase in protein stability. This program predicts the stability of a protein after mutation. A protein stability test was then run on the pathogenic SNPs. Substantially deleterious nsSNPs are those that reduce protein stability.

### 2.6. Prediction of the Structural Effect of High-Risk nsSNPs on Human Dectin-1 Protein

Project HOPE was used to analyze the predicted effect of the nsSNPs or point mutations on the structure of the *Dectin-1* protein. It is a web server that identifies the structural effects of point mutations in a protein sequence [23]. We used NP_922938.1 (the NCBI Reference Sequence Code of *CLEC7A*) and the 25 SNPs individually as the input. Missense 3D [24] confirmed the results’ stringency and accuracy. Missense 3D predicts the effect of an amino acid substitution on protein structure. This study evaluated structural features used by the Missense3D web server, including disulfide bond breakage and buried H-bond breakage.

### 2.7. Prediction of the Post-Translational Site’s Modification

MusiteDeep is an online tool for visualizing PTM sites in protein sequences (https://www.musite.net/ (accessed on 7 April 2023)). MusiteDeep determines post-translational modifications such as phosphorylation, glycosylation, ubiquitination, sumoylation, acetyl-lysine, methylation, pyrrolidone carboxylic acid, palmitoylation, and hydroxylation [25]. The input query for MusiteDeep was the FASTA format of the *Dectin-1* protein sequence.

### 2.8. Analysis of 5′ and 3′ UTR Non-Coding SNPs

The ENSEMBL database was used to investigate non-coding regions [26]. We filtered out SNPs from the 5′ and 3′ regions, and a minor allelic frequency (MAF) value of ≤0.001 was selected. Regulome DB was used to relate SNPs to regulatory elements of the human genome [27]. It also provides chip data, chromatin states, motif information, and ranking based on DNA binding, if available. The PolymiRTS database was utilized to predict if there is DNA variation in miRNA target sites in the 3′ UTR region [28]. The results are presented in four classes: D, O, C, and N, along with context and conservation scores, miR IDs, and target miR sites for miRs.

### 2.9. Determining CLEC7A Protein-Protein Interaction (PPI) Network Analysis

Protein-protein interactions are essential for regulating and executing a protein’s biological functions. STRING (Search Tool for the Retrieval of Interacting Genes/Proteins) predicts the top ten proteins interacting with the query gene by predicting their interactions with the *CLEC7A* protein (https://string-db.org (accessed on 7 April 2023)). The STRING algorithm predicts the interaction partners of a protein based on gene fusion, co-expression, function, and experimental data. Each interacting protein is scored from 0 to 1, where 0 indicates the lowest interaction and 1 indicates the highest interaction [29]. The *CLEC7A* FASTA protein sequence was submitted as input. All predicted interactions associated with confidence scores are included in the output.

### 2.10. Functional and Pathway Enrichment Analysis Using STRING

The functional analysis involved annotating and enriching proteins in the network based on their functional properties. The most common enrichment analysis is based on gene ontology (GO) terms (i.e., biological processes, molecular functions, and cellular components) and pathways. In order to interpret the physical function of a network, functional analysis is necessary. Gene Ontology (GO) and Kyoto Encyclopedia of Genes and Genomes (KEGG) pathway enrichment analyses were conducted using STRING [29].

## 3. Results

### 3.1. Retrieving nsSNPs of Dectin-1

*Dectin-1* SNPs were retrieved using the NCBI dbSNP database. It comprised 3904 SNPs, of which 216 were missense (nsSNP), 758 non-coding transcripts, 247 codings, 93 synonymous, 3858 intronic, 1 inframe insertion, and 2 inframe deletions—regarding clinically significant, 1 pathogenic, 6 benign, and 8 likely benign. For our current study, we only selected nsSNPs in the coding and non-coding regions (3UTR and 5UTR) for further analyses.

### 3.2. Prediction of Pathogenicity of nsSNPs

Results from predictSNP software were compared to predict the pathogenicity of *Dectin-1* nsSNPs precisely and label the highly risky deleterious SNPs that can significantly alter the structure or function. Out of 247 nsSNPs, 27 were predicted to be deleterious SNPs in all computational algorithms (Table 1).

### 3.3. Identification of the Domains of Dectin-1

**Figure 2:**
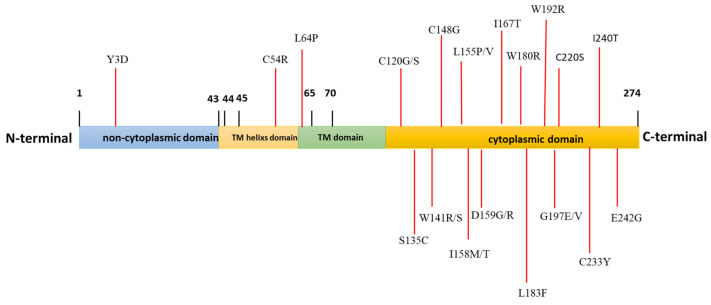
Domain identification of *Dectin-1* protein using InterPRO server and the position of nsSNPs in each domain. The *Dectin-1* protein (1–274 aa), non-cytoplasmic domain (1–44 aa), TMhelixs domain (43–65 aa), transmembrane (45–70 aa), and cytoplasmic domain (71–274 aa).

Using the InterPro tool, four functional domains of *Dectin-1* were predicted. The domains were the non-cytoplasmic domain (1–44), TMhelix domain (43–65), transmembrane (45–70), and cytoplasmic domain (71–274) (Figure 2 and Figure S1).

### 3.4. Structural Analysis

#### 3.4.1. Determination of Protein Structural Stability (I-Mutant 2.0 Analysis)

We introduced the 28 nsSNPs into the *Dectin-1* protein using the I-Mutant 2.0 tool. The outcome revealed that 25 out of 28 deleterious nsSNPs decreased stability (Table 2). The DDG value was calculated from the mutated protein’s unfolding Gibbs free energy value minus the wild type’s unfolding Gibbs free energy value (Kcal/mol). DDG/DDG was positive for D13Y, S22F, and S117F and negative for the remaining nsSNPs run by this tool.

#### 3.4.2. Evolutionary Conservation Analysis

**Figure 3:**
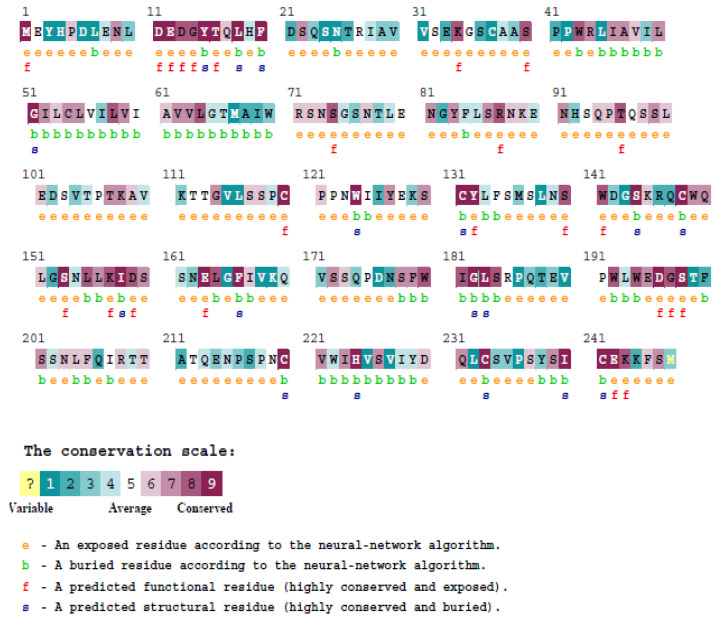
Evolutionary conservancy of amino acids in *Dectin-1* analyzed by Consurf.

The ConSurf web server [19,20] determined the evolutionary conservancy of amino acid residues in the native *Dectin-1*. Of the 25 high-risk nsSNPs of the *Dectin-1* protein, we found that C120G, C120S, W141R, W141S, D159G, D159R, G197E, G197V, D13Y, and E242G are exposed and functional according to the neural-network algorithm. Whereas, C220S, C233Y, I240T, L183F, I158M, I158T, and C148G residues are buried and structural (Figure 3 and Figure S1).

#### 3.4.3. Project HOPE Results for Comparing Wild-Type and Mutant Amino Acid Properties

Project HOPE evaluated the differences in wild-type and mutant amino acids in terms of size, charge, hydrophobicity value, and possible interactions that mutated residues might induce. All 25 nsSNPs have a deleterious effect on protein structure (Table S2). *Dectin-1* protein amino acid residues were altered in size, charge, and hydrophobicity at their respective positions. Amino acid residue sizes were changed from large to small, and the charge was observed to be lost or gained. The loss of hydrogen bonds caused by nsSNPs also increased or decreased hydrophobicity in the protein. As a result of these changes, the protein cannot fold correctly.

#### 3.4.4. Missense 3D Results

Missense 3D predicted that 7 out of 25 nsSNPs have structural damage for the *Dectin-1* protein. C120S, C148G, C233Y, and C220S have disulfide breakage; W141R has buried H-bond breakage; W141S has buried hydrophilic introduced; buried charge introduced; L155P has buried pro introduced; buried H-bond breakage; and W180R has buried hydrophilic introduced; buried charge introduced (Table 3).

### 3.5. Analysis of 5′ and 3′ UTR Non-Coding SNPs

After setting the MAF filter to more than or equal to 0.001, 27 SNPs in the 3UTR and 2 SNPs in the 5UTR were found in the Ensemble database. The gene variants of *Dectin-1* with transcript ID ENST00000304084.8 (Table 4. In Regulome DB, only the SNPs with a ranking < 4 were considered, and two SNPs, rs536465890 and rs527258220, were chosen for 5UTR. No SNPs were found for 3UTR. The rankings, along with the probability score, are given in Table 4.

### 3.6. PolymiRTS Analysis

SNPs in the PolymiRTS database were classified into four functional groups: (1) D describes the disruption of a conserved miRNA site; (2) *n* describes the disruption of a non-conserved miRNA site; (3) C denotes the creation of a new miRNA site; and (4) O denotes the absence of a determination of an ancestral allele. The results show that all of the miRNA target sites for miRNA predicted to be disrupted by SNPs in *Dectin-1* were obtained from CLASH experimental data (*n*). No nsSNPs were obtained when filtered, considering the functional classes of C and D and conservation scores of 10 (Table S3).

### 3.7. Protein-Protein Interactions Analysis and Functional Enrichments Analysis

**Figure 4:**
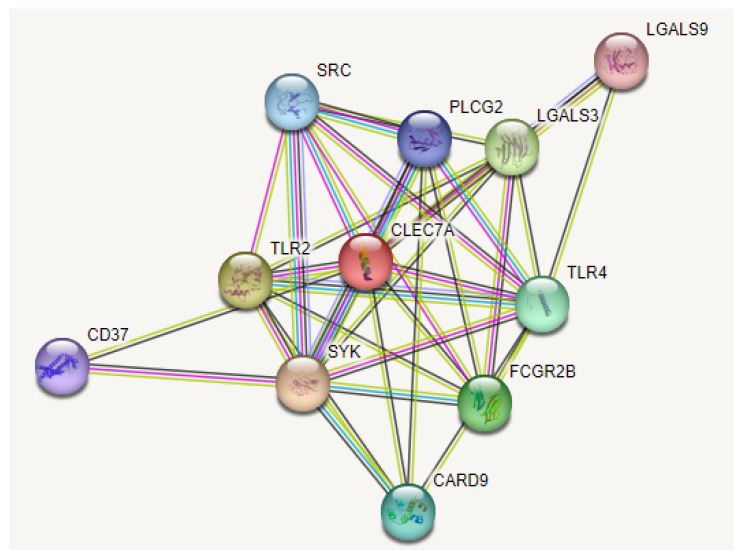
Protein interaction network of *CLEC7A* protein.

A STRING interaction analysis revealed that the *CLEC7A* gene is involved in many molecular and biological processes. The *CLEC7A* has high-confidence interactions with predicted functional partners, including spleen-associated tyrosine kinase (SYK), which mediates signal transduction downstream of a variety of transmembrane receptors, including classical immunoreceptors like the B-cell receptor; Toll-like receptor 2 (TLR2), which cooperates with LY96 to mediate the innate immune response to bacterial lipoproteins and other microbial cell wall components; Galectin-3; Galactose-specific lectin (LGALS3), which binds IgE; and Fc gamma receptor IIb (FCGR2B), aggregated immunoglobulins gamma. It is involved in various effector and regulatory functions, such as phagocytosis of immune complexes and modulation of antibody production by B-cells. Toll-like receptor 4 (TLR4) cooperates with LY96 and CD14 to mediate the innate immune response to bacterial lipopolysaccharide (LPS). Caspase recruitment domain-containing protein 9 (CARD9) is an adapter protein that plays a crucial role in the innate immune response to several intracellular pathogens, such as *C. albicans* and *L. monocytogenes*. Proto-oncogene tyrosine-protein kinase (SRC), a non-receptor protein tyrosine kinase that is activated following the engagement of many different classes of cellular receptors, including immune response receptors; 1-phosphatidylinositol 4,5-bisphosphate phosphodiesterase gamma-2 (PLCG2), which is a crucial enzyme in transmembrane signaling; C2 domain containing phospholipases; leukocyte antigen CD37 (CD37); and Galectin 9 (LGALS9), which stimulates bactericidal activity in infected macrophages by causing macrophage activation. All interactions are shown in Figure 4. Functional enrichments of the *Dectin-1* network, including KEGG and GO, demonstrate that the *Dectin-1* protein is involved in multiple immune signaling pathways [29] (Table S4).

## 4. Discussion

SNPs are considered among the most significant risk factors associated with many diseases. The presence of SNPs within the protein-coding and non-coding regions can adversely affect the function and conformation of the protein. This study aimed to determine which of the most damaging nsSNP variants may affect the functionality of *Dectin-1*. To our knowledge, no comprehensive in silico analysis has been performed to predict deleterious nsSNPs in coding and non-coding regions. In 2017, only three mutations of the 91 nsSNPs reported were predicted to be responsible for altering the protein structure of *Dectin-1*. The three mutations predicted were I223S (rs16910527), I158T 306 (rs138005591), and D159G (rs758623997) [30]. This study also detected the same mutations among other high-risk mutations using two additional in-silico algorithms (PolyPhen-1 and PredictSNP), two structural servers (I-Mutant3.0 and Project Hope), protein-protein interactions analysis, and functional and pathway enrichment analysis using STRING. Raman et al. proposed the consideration of the three non-synonymous SNPs in the *Dectin-1* gene as a risk assessment against fungal infections.

Effectively distinguishing between harmful and tolerated nsSNP types involves successive filtering using several disease-associated bioinformatics tools. Six functional tools (MAPP, PhD-SNP, PolyPhen-1, PolyPhen-2, SIFT, and SNAP) and two structural servers (I-Mutant3.0 and Project Hope) were applied to determine whether the identified nsSNPs were harmful or benign. Among the 247 nsSNPs found in the SNPs from the NCBI database, 25 nsSNPs with distinct mutations (C54R, L64P, C120G, C120S, S135C, W141R, W141S, C148G, L155P, L155V, I158M, I158T, D159G, D159R, I167T, W180R, L183F, W192R, G197E, G197V, C220S, C233Y, I240T, E242G, and Y3D) were identified as likely to be harmful, deleterious, or disease-causing, based on the outcomes of 8 software tools. Other previously reported FSNPs included Y238X, which has been implicated in increasing the risk of Candida species colonization in the gastrointestinal tract of immunosuppressed patients without increasing the risk of candidemia. In diabetic populations, FSNPs (C/G SNP at position -44) are protective in secreting antifungal peptides by epithelial b-defensins.

InterPro software was used to determine the location of these nsSNPs on different domains of *Dectin-1*. It revealed 28 nsSNPs in three protein domains, of which two were positioned in the non-cytoplasmic domain. Nine nsSNPs were located in the TMhelix/transmembrane domain, and the rest (16 nsSNPs) were present in the cytoplasmic domain containing the immunoreceptor tyrosine-based activation motif-like sequence (ITAM-like). This ITAM mediates a ligand-induced signaling response’s activation by interacting with the SKY protein and producing various immune modulators, suggesting the presence of any of the nsSNPs could disrupt the immune signaling pathways.

According to the ConSurf server, the evolutionary model of macromolecules is interpreted to demonstrate the importance of highly conserved regions in the function and conformation of macromolecules. Furthermore, the functional regions of proteins are often conserved and associated with various functions, including catalysis, interaction, and binding [27,28]. Our analysis of the ConSurf web server found 26 highly deleterious nsSNPs located in highly conserved regions. Thus, increasing the risk of inactivating *Dectin-1* and reducing antifungal effects. This suggests that these variants may alter the function and conformation of the protein. The conformational changes of the *Dectin-1* protein during biomolecular interactions are vital for executing its function. In addition, nsSNPs can lead to aberrant conformations, which may inactivate their antifungal properties [31]. Hence, it is crucial to determine the effect of deleterious nsSNPs in *Dectin-1* and their association with various diseases.

The stability of proteins is vital to their structural and functional activity [32]. The I-Mutant results showed that 25 nsSNPs decreased the stability of the protein. There are several consequences of changes in protein stability, including misfolding, degradation, or aberrant conglomeration of proteins [33].

There is a high risk of pathology when mutations occur in or near some special amino acids that contribute to functional and spatial conformation. A missense mutation results in amino acid substitutions, resulting in changes in amino acid size, charge, and hydrophobicity, which may disrupt the folding and interaction of proteins. The analysis from HOPE indicates that mutations lead to either loss of interactions or structural changes, particularly in transmembrane domains. In addition, introducing or losing charge or hydrophobicity would result in repulsion, misfolding, or loss of interactions. The Hope project results demonstrated that all 25 nsSNPs have a deleterious effect on the *Dectin-1* structure and consequently contribute to the loss of its function and anti-fungal or microbial properties. Furthermore, the Missense 3D tool predicted the consequences of the 28 structural nsSNPs and showed that only seven nsSNPs deleteriously affect the structural conformation of the *Dectin-1* protein. 

Biological processes and disease development depend on the regulation of PTMs. Among the most harmful nsSNPs are those that alter PTM sites and are associated with the disease. We found none of the PTM sites we identified were affected by our nsSNPs using MusiteDeep software.

miRNA binds to mRNA and inhibits mRNA translation through mRNA degradation to regulate protein production. A 3UTR nsSNP in the gene can modify or disrupt mRNA target sites, altering miRNA-mRNA interactions and possibly leading to abnormal gene expression [34]. In this regard, non-coding SNPs can interfere with normal gene expression and protein synthesis regulation. According to the PolymiRTS database, the D and C classes with high conservation and negative context scores have the highest functionally probable effects. Class D refers to disrupting a conserved site, while class C refers to creating a new site [28]. PolymiRTS analysis showed that no non-coding SNPs could alter the miRNA binding sites in the 3UTR of *Dectin-1*. In Regulome DB, only the SNPs with a ranking *<* 4 were considered. No nsSNPs were found for 3UTR. The rankings, along with the probability score, are given. According to Regulome DB, among the non-coding SNPs, rs536465890 and rs527258220 showed the best results for 5UTR. 

From the STRING protein-protein interaction analysis, *Dectin-1* was predicted to have strong interactions with SYK, TLR2, LGALS3, FCGR2B, TLR4, CARD9, SRC, PLCG2, LGALS9, and CD37. The STRING interaction result was further validated by using KEGG pathways for *Dectin-1*. Interestingly, the same set of proteins was involved in an immune signaling pathway. STRING analysis demonstrated that SYK, TLR4, CARD9, and TLR2 are involved in vulvovaginal candidiasis and infectious disease pathways. Therefore, any of the identified nsSNPs may change the *Dectin-1* protein’s function.

Studies have shown that a stop codon polymorphism causes inadequate *Dectin-1* receptor activity, possibly enhancing vulnerability to invasive aspergillosis (IA) [35]. Cunha et al. and Sainz et al.’s analysis of bone marrow transplant patients and donors discovered that the *Dectin-1* receptor variant is a risk factor for IA in high-risk patients. Both groups had an associated degree of significance (OR = 4.91, 95% CI = 1.52–15.9, *p* = 0.05). Sainz et al. further demonstrated an increase in the proportion of these polymorphism-carrying galactomannan-positive patients. Chai et al. and Smith et al. also confirmed the relationship between variation in the gene encoding *Dectin-1* and IA. Recently, it has been discovered that several polymorphisms in genes that encode innate immunity-related proteins enhance susceptibility to Aspergillus infections [31]. The likelihood that a patient has more than one polymorphism and hence has a high vulnerability to developing IA is essential. Chai et al. indicated a strong correlation between the emergence of IA and genetic variations. Although *Dectin-1* is one of the main phagocytic receptors in macrophages for fungal infections, other SNPs in other genes have also been reported to affect human-fungal recognition and interaction. These genes include mannose-binding lectin (MBL) 2, toll-like receptors (TLRs 1, 2, 3, 4), CARD9 caspase recruitment domain-containing protein 9, IL-4, and b-Defensin-1 [36].

## 5. Conclusions

In this study, we have identified several novel nsSNPs in the *Dectin-1* gene that may be considered risk targets against fungal infections. The frequency of such mutations in local populations can be determined in the future through epidemiological research, as can whether these mutations are associated with fungal infections. This study could aid in identifying populations more susceptible to developing fungal infections by identifying genetic variations associated with IA or Candidiasis.

## Figures and Tables

**Table 1:** High-risk nsSNPs identified by seven in silico programs.

SNP Id	AA Change	PredictSNP, MAPP, PhD-SNP, SIFT, SNAP	Polyphen1, Polyphen2
rs756166982	D13Y	Deleterious	Damaging
rs759032825	S22F	Deleterious	Damaging
rs775715931	C54R	Deleterious	Damaging
rs781427660	L64P	Deleterious	Damaging
rs112345533	S117F	Deleterious	Damaging
rs1013923644	C120G	Deleterious	Damaging
rs1156591610	C120S	Deleterious	Damaging
rs1422790966	S135C	Deleterious	Damaging
rs761503556	W141R	Deleterious	Damaging
rs369482852	W141S	Deleterious	Damaging
rs746386372	C148G	Deleterious	Damaging
rs1256594278	L155P	Deleterious	Damaging
rs747442135	L155V	Deleterious	Damaging
rs1346068120	I158M	Deleterious	Damaging
rs138005591	I158T	Deleterious	Damaging
rs758623997	D159G	Deleterious	Damaging
rs1302972586	D159R	Deleterious	Damaging
rs1262393046	I167T	Deleterious	Damaging
rs1221428821	W180R	Deleterious	Damaging
rs140318683	L183F	Deleterious	Damaging
rs1307651895	W192R	Deleterious	Damaging
rs1255198388	G197E	Deleterious	Damaging
rs1255198388	G197V	Deleterious	Damaging
rs1267664350	C220S	Deleterious	Damaging
rs141153031	C233Y	Deleterious	Damaging
rs1219119993	I240T	Deleterious	Damaging
rs1458236572	E242G	Deleterious	Damaging

**Table 2:** Effect of nsSNPs on protein stability predicted by I-MUTANT 2.0.

SNP Id	AA Change	Protein Domains	Position	I-Mutant	RI	DDG-Free Energy Change Value (kcal/mol)
rs562749381	Y3D	non-cytoplasmic domain	3	Decrease	6	−1.08
rs756166982	D13Y		13	Increase	5	0.07
rs759032825	S22F		22	Increase	4	0.27
rs775715931	C54R	TMhelixs domain/transmembrane domain	54	Decrease	2	−0.21
rs781427660	L64P		64	Decrease	7	−1.52
rs112345533	S117F	cytoplasmic domain	117	Increase	2	0.1
rs1013923644	C120G		120	Decrease	8	−1.38
rs1156591610	C120S		120	Decrease	7	−0.87
rs1422790966	S135C		135	Decrease	6	−0.82
rs761503556	W141R		141	Decrease	8	−1.02
rs369482852	W141S		141	Decrease	9	−1.56
rs746386372	C148G		148	Decrease	7	−1.02
rs1256594278	L155P		155	Decrease	1	−1.34
rs747442135	L155V		155	Decrease	6	−1.31
rs1346068120	I158M		158	Decrease	6	−1.48
rs138005591	I158T		158	Decrease	7	−2.15
rs758623997	D159G		159	Decrease	7	−1.5
rs1302972586	D159R		159	Decrease	4	−0.59
rs1262393046	I167T		167	Decrease	9	−2.4
rs1221428821	W180R		180	Decrease	8	−1.15
rs140318683	L183F		183	Decrease	5	−1.03
rs1307651895	W192R		192	Decrease	7	−1
rs1255198388	G197E		197	Decrease	3	−0.42
rs1255198388	G197V		197	Decrease	5	−0.35
rs1267664350	C220S		220	Decrease	8	−0.93
rs141153031	C233Y		233	Decrease	3	−0.43
rs1219119993	I240T		240	Decrease	9	−2.06
rs1458236572	E242G		242	Decrease	7	−1.21

**Table 3:** Missense 3D analysis of the structural impact of seven missense nsSNPs in *Dectin-1* protein highlighted in gray. The other residues were not determined in our study.

Uniprot Position	PDB/Model Position	Residue Wild-Type	Residue Mutant	Missense3D Prediction	Structural Damage Predicted
120	120	CYS	SER	Damaging	Disulphide breakage
133	133	LEU	PRO	Damaging	Secondary structure altered; Disallowed phi/psi
141	141	TRP	SER	Damaging	Buried H-bond breakage
141	141	TRP	ARG	Damaging	Buried hydrophilic introduced; Buried charge introduced
148	148	CYS	GLY	Damaging	Disulphide breakage
155	155	LEU	PRO	Damaging	Buried Pro introduced; Buried H-bond breakage
157	157	LYS	ARG	Damaging	Buried/exposed switch
163	163	GLU	LYS	Damaging	Buried/exposed switch
180	180	TRP	ARG	Damaging	Buried hydrophilic introduced; Buried charge introduced
184	184	SER	PHE	Damaging	Buried H-bond breakage
185	185	ARG	GLN	Damaging	Buried charge replaced; Buried salt bridge breakage
185	185	ARG	CYS	Damaging	Buried charge replaced; Buried salt bridge breakage
185	185	ARG	HIS	Damaging	Buried H-bond breakage; Buried salt bridge breakage
188	188	THR	PRO	Damaging	Disallowed phi/psi
191	191	PRO	SER	Damaging	Secondary structure altered
191	191	PRO	LEU	Damaging	Secondary structure altered
216	216	PRO	THR	Damaging	Secondary structure altered
220	220	CYS	SER	Damaging	Disulphide breakage
233	233	CYS	TYR	Damaging	Disulphide breakage
238	238	TYR	HIS	Damaging	Buried/exposed switch
239	239	SER	ASN	Damaging	Buried H-bond breakage

**Table 4:** Regulome DB data for non-coding SNPs of *Dectin-1* 3UTR and 5UTR.

*Dectin-1*	Chromosome Location	dbSNP IDs	Rank	Score
3UTR	chr12:10269383..10269384	rs568706240	6	0.16346
	chr12:10269395..10269396	rs531257836	6	0.1131
	chr12:10269470..10269471	rs553392700	6	0
	chr12:10269472..10269473	rs566870430	7	0.18412
	chr12:10269515..10269516	rs182562001	7	0.18412
	chr12:10269552..10269553	rs555302379	6	0.29006
	chr12:10269556..10269557	rs575479504	6	0.08083
	chr12:10269718..10269719	rs185282370	7	0.18412
	chr12:10269727..10269728	rs577169427	6	0.4855
	chr12:10269916..10269917	rs542384129	5	0.39056
	chr12:10269919..10269920	rs562187863	5	0
	chr12:10270059..10270060	rs143144453, rs535611004	6	0.182
	chr12:10270088..10270089	rs557589771	7	0.18412
	chr12:10270148..10270149	rs187544967	6	0.16346
	chr12:10270339..10270340	rs573661932	7	0.18412
	chr12:10270414..10270415	rs562608492	7	0.18412
	chr12:10270458..10270459	rs193279976	7	0.18412
	chr12:10270704..10270705	rs560325803	5	0.13454
	chr12:10270713..10270714	rs529384924	5	0
	chr12:10270739..10270740	rs569018735	6	0.80633
	chr12:10270770..10270771	rs537612920	6	0.41186
	chr12:10270969..10270970	rs576227282	6	0.17931
	chr12:10270980..10270981	rs564703740	6	0.40391
	chr12:10271102..10271103	rs141153031	5	0.13454
5UTR	chr12:10282801..10282802	rs536465890	2b	0.64862
	chr12:10282827..10282828	rs527258220	2b	0.67017
	chr12:10282834..10282835	rs143367407	4	0.60906

## Data Availability

All data is provided in the Supplementary Materials. All data base links are shared in the Section 2.

## Supplementary Materials

The following supporting information can be downloaded at: https://www.mdpi.com/article/10.3390/diagnostics13101785/s1, Figure S1: Domain identification of *Dectin-1* protein using InterPRO server; Table S1: Evolutionary conservancy of amino acids in *Dectin-1* analyzed by Consurf; Table S2: Structural effect of 25 nsSNPs over *Dectin-1* protein using Project Hope. Table S3: miRNA binding site prediction of non-coding SNPs in *Dectin-1* protein through PolymiRTS. Table S4: Functional enrichments analysis of *Dectin-1* network.
