# Supplementary material for: Comprehensive In Silico Characterization of the Coding and Non-Coding SNPs in Human Dectin-1 Gene with the Potential of High-Risk Pathogenicity Associated with Fungal Infections
Source: Diagnostics (Basel). 2023 May 18;13(10):1785. doi: 10.3390/diagnostics13101785 (PMC10217694; doi:10.3390/diagnostics13101785)
Supplement: Supplementary file 1 [file diagnostics-13-01785-s001.zip › diagnostics-2363026-supplementary.pdf]

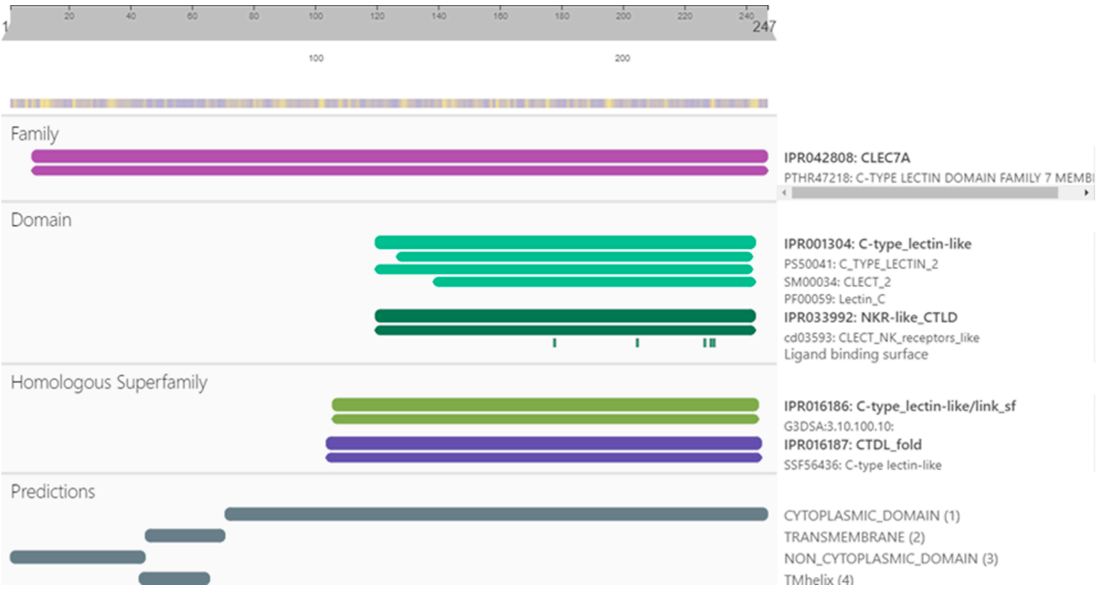

Figure S1. Domain identification of Dectin-1 protein using InterPRO server. IPR016186 indicates the Dectin-1 protein (1-274aa), non-cytoplasmic domain (1-44), TMhelixs domain (43-65), transmembrane (45-70), and cytoplasmic domain (71-274).

Table S1. Evolutionary conservancy of amino acids in Dectin-1 analyzed by Consurf

SNP Id	Residue and Position	Domains	Conservation Score	Prediction
rs562749381	Y3D	non-cytoplasmic domain	1	An exposed residue, according to the neural-network algorithm
rs756166982	D13Y		8	A predicted functional residue (highly conserved and exposed)(E,F)
rs759032825	S22F		8	e - An exposed residue according to the neural-network algorithm.
rs775715931	C54R	TMhelixs domain/ transmembrane domain	8	b - A buried residue according to the neural-network algorithm.
rs781427660	L64P		9	b - A buried residue according to the neural-network algorithm.
rs112345533	S117F	cytoplasmic domain	7	e - An exposed residue according to the neural-network algorithm.
rs1013923644	C120G		9	A predicted functional residue (highly conserved and exposed)(E,F)
rs1156591610	C120S		9	A predicted functional residue (highly conserved and exposed)(E,F)
rs1422790966	S135C			Insufficient data - the calculation for this site was
rs761503556	W141R		9	A predicted functional residue (highly conserved and exposed)(E,F)
rs369482852	W141S		9	A predicted functional residue (highly conserved and exposed)(E,F)
rs746386372	C148G		9	A predicted structural residue (highly conserved and buried (S)
rs1256594278	L155P		8	b - A buried residue according to the neural-network algorithm
rs747442135	L155V		8	b - A buried residue according to the neural-network algorithm
rs1346068120	I158M		9	s - A predicted structural residue (highly conserved and buried).
rs138005591	I158T		9	s - A predicted structural residue (highly conserved and buried).
rs758623997	D159G		8	A predicted functional residue (highly conserved and exposed).
rs1302972586	D159R		8	A predicted functional residue (highly conserved and exposed).
rs1262393046	I167T		7	b - A buried residue according to the neural-network algorithm.
rs1221428821	W180R		7	b - A buried residue according to the neural-network algorithm.
rs140318683	L183F		9	s - A predicted structural residue (highly conserved and buried).
rs1307651895	W192R		8	b - A buried residue according to the neural-network algorithm.
rs1255198388	G197E		8	A predicted functional residue (highly conserved and exposed)
rs1255198388	G197V		8	A predicted functional residue (highly conserved and exposed)

rs1267664350	C220S	9	s - A predicted structural residue (highly conserved and buried).
rs141153031	C233Y	9	s - A predicted structural residue (highly conserved and buried).
rs1219119993	I240T	9	s - A predicted structural residue (highly conserved and buried).
rs1458236572	E242G	9	f - A predicted functional residue (highly conserved and exposed).

Table S2. Structural effect of 25 nsSNPs over *Dectin-1* protein using Project Hope

Residue	AA change	Structure	AA Properties
rs562749381	Y3D	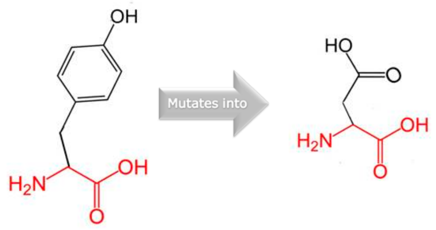	• There is a difference in charge between the wild-type and substituted amino acid.<br>• The mutation introduces a charge, this can cause **repulsion of ligands** or other residues with the same charge.<br>• The wild-type and substituted amino acids differ in size.<br>• The mutant residue is smaller, this might lead to loss of interactions.<br>• The hydrophobicity of the wild-type and mutant residue differs.<br>• Hydrophobic interactions, either in the core of the protein or on the surface, will be lost.
rs775715931	C54R	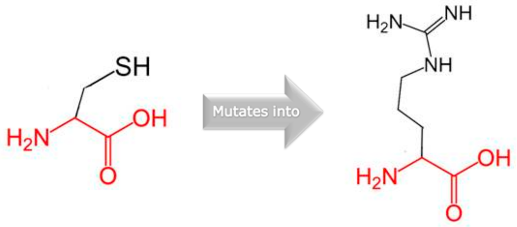	• There is a difference in charge between the wild-type and substituted amino acid.<br>• The mutation introduces a charge, this can cause **repulsion of ligands** or other residues with the same charge.<br>• The wild-type and substituted amino acids differ in size.<br>• The mutant residue is bigger, this might lead to bumps.<br>• The hydrophobicity of the wild-type and mutant residue differs.<br>• Hydrophobic interactions, either in the core of the protein or on the surface, will be lost.
rs781427660	L64P	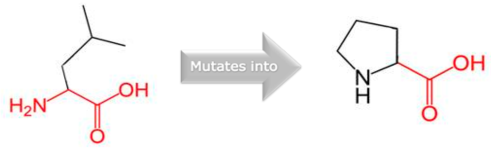	• The wild-type and substituted amino acids differ in size.<br>• The mutant residue is smaller than the wild-type residue.<br>• This will cause a possible **loss of external interactions**.

rs1013923644	C120G	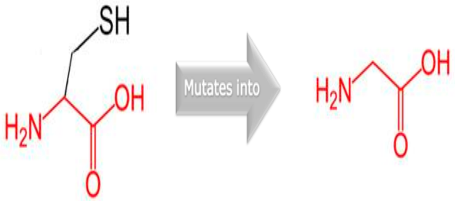	• The wild-type and substituted amino acids differ in size.<br>• The mutant residue is smaller than the wild-type residue.<br>• The mutation will cause an empty space in the core of the protein.<br>• The hydrophobicity of the wild-type and mutant residue differs.<br>• The mutation will cause loss of hydrophobic interactions in the core of the protein.
rs1156591610	C120S	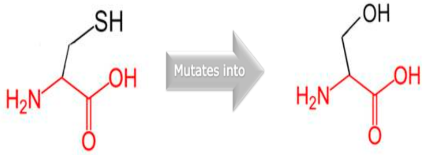	• The hydrophobicity of the wild-type and mutant residue differs.<br>• The mutation will cause loss of hydrophobic interactions in the core of the protein.
rs1422790966	S135C	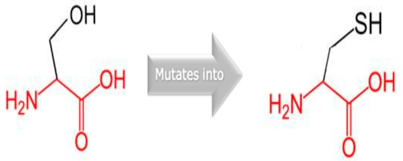	• The hydrophobicity of the wild type and mutant residue differs.<br>• The mutation will cause loss of hydrogen bonds in the core of the protein and as a result disturb correct folding.

rs761503556	W141R	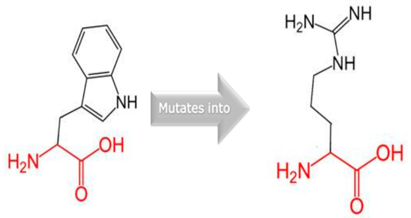	• There is a difference in charge between the wild-type and substituted amino acid.<br>• The mutant residue introduces a charge in a buried residue which can lead to protein folding problems.<br>• The wild-type and substituted amino acids differ in size.<br>• The mutant residue is smaller than the wild-type residue.<br>• The mutation will cause an empty space in the core of the protein.<br>• The hydrophobicity of the wild-type and mutant residue differs.<br>• The mutation will cause loss of hydrophobic interactions in the core of the protein.
rs369482852	W141S	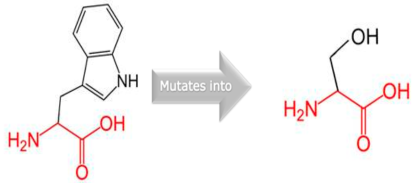	• The wild-type and substituted amino acids differ in size.<br>• The mutant residue is smaller than the wild-type residue.<br>• The mutation will cause an empty space in the core of the protein.<br>• The hydrophobicity of the wild-type and mutant residue differs.<br>• The mutation will cause loss of hydrophobic interactions in the core of the protein.
rs746386372	C148G	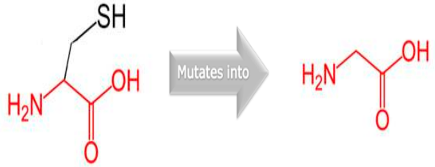	• The wild-type and substituted amino acids differ in size.<br>• The mutant residue is smaller than the wild-type residue.<br>• The mutation will cause an empty space in the core of the protein.<br>• The hydrophobicity of the wild-type and mutant residue differs.<br>• The mutation will cause loss of hydrophobic interactions in the core of the protein.
rs1256594278	L155P	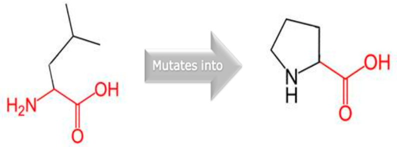	• The wild-type and substituted amino acids differ in size.<br>• The mutant residue is smaller than the wild-type residue.<br>• The mutation will cause an empty space in the core of the protein.
rs747442135	L155V	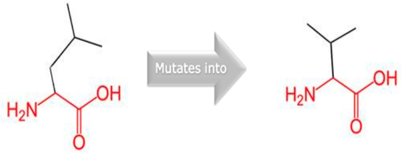	• The wild-type and substituted amino acids differ in size.<br>• The mutant residue is smaller than the wild-type residue.<br>• The mutation will cause an empty space in the core of the protein.

rs1346068120	I158M	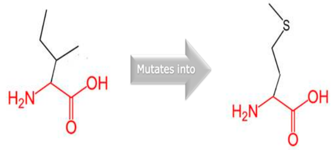	• The wild-type and substituted amino acids differ in size.<br>• The mutant residue is bigger than the wild-type residue.<br>• The wild-type residue was buried in the core of the protein. The mutant residue is bigger and probably will not fit.
rs138005591	I158T	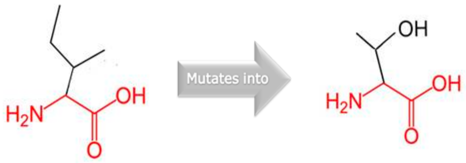	• The wild-type and substituted amino acids differ in size.<br>• The mutant residue is smaller than the wild-type residue.<br>• The mutation will cause an empty space in the core of the protein.<br>• The hydrophobicity of the wild-type and mutant residue differs.<br>• The mutation will cause loss of hydrophobic interactions in the core of the protein.
rs758623997	D159G	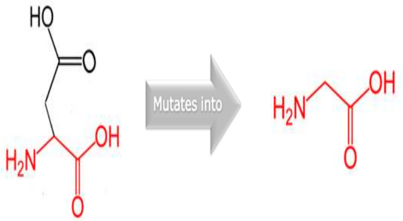	• There is a difference in charge between the wild-type and substituted amino acid.<br>• The charge of the wild-type residue is lost by this mutation. This can cause loss of interactions with other molecules.<br>• The wild-type and substituted amino acids differ in size.<br>• The mutant residue is smaller than the wild-type residue.<br>• This will cause a possible loss of external interactions.<br>• The hydrophobicity of the wild-type and mutant residue differs.
rs1302972586	D159R	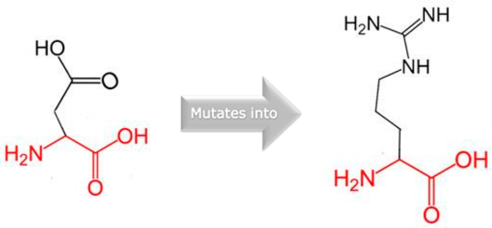	• There is a difference in charge between the wild-type and substituted amino acid.<br>• The mutation introduces the opposite charge at this position. This possibly disrupts contacts with other molecules.<br>• The wild-type and substituted amino acids differ in size.<br>• The mutant residue is bigger than the wild-type residue.<br>• The residue is located on the surface of the protein, mutation of this residue can disturb interactions with other molecules or other parts of the protein.

rs1262393046	I167T	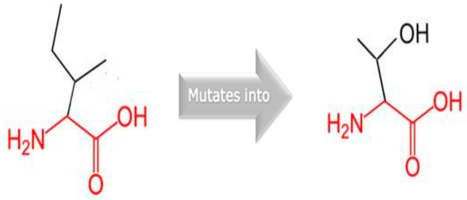	• The wild-type and substituted amino acids differ in size.<br>• The mutant residue is smaller than the wild-type residue.<br>• The mutation will cause an empty space in the core of the protein.<br>• The hydrophobicity of the wild-type and mutant residue differs.<br>• The mutation will cause loss of hydrophobic interactions in the core of the protein.
rs1221428821	W180R	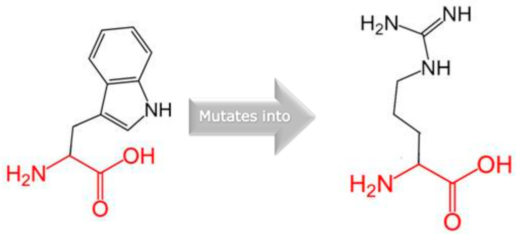	• There is a difference in charge between the wild-type and substituted amino acid.<br>• The mutant residue introduces a charge in a buried residue which can lead to protein folding problems.<br>• The wild-type and substituted amino acids differ in size.<br>• The mutant residue is smaller than the wild-type residue.<br>• The mutation will cause an empty space in the core of the protein.<br>• The hydrophobicity of the wild-type and mutant residue differs.<br>• The mutation will cause loss of hydrophobic interactions in the core of the protein.
rs140318683	L183F	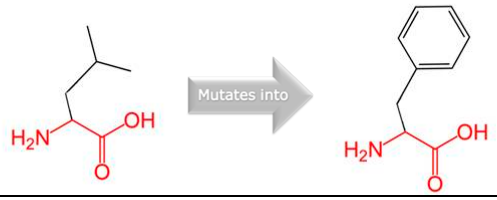	• The wild-type and substituted amino acids differ in size.<br>• The mutant residue is bigger than the wild-type residue.<br>• The wild-type residue was buried in the core of the protein. The mutant residue is bigger and probably will not fit.
rs1307651895	W192R	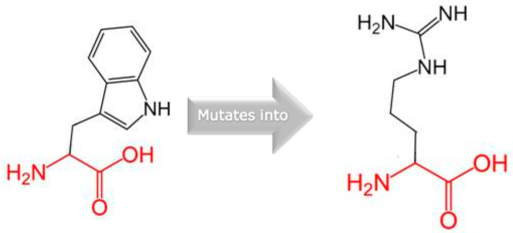	• There is a difference in charge between the wild-type and substituted amino acid.<br>• The mutation introduces a charge at this position, this can cause repulsion between the mutant residue and neighboring residues.<br>• The wild-type and substituted amino acids differ in size.<br>• The mutant residue is smaller than the wild-type residue.<br>• This will cause a possible loss of external interactions.<br>• The hydrophobicity of the wild-type and mutant residue differs.<br>• The mutation might cause loss of hydrophobic interactions with other molecules on the surface of the protein.

rs1255198388	G197E	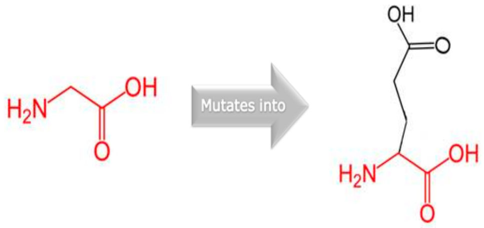	➢ There is a difference in charge between the wild-type and substituted amino acid.<br>➢ The mutation introduces a charge at this position, this can cause repulsion between the mutant residue and neighboring residues.<br>➢ The wild-type and substituted amino acids differ in size.<br>➢ The mutant residue is bigger than the wild-type residue.<br>➢ The residue is located on the surface of the protein, mutation of this residue can disturb interactions with other molecules or other parts of the protein.<br>➢ The torsion angles for this residue are unusual. only glycine is flexible enough to make these torsion angles, mutation into another residue will force the local backbone into an incorrect conformation and will disturb the local structure.
rs1255198388	G197V	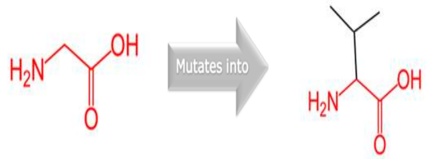	➢ The wild type and substituted amino acids differ in size.<br>➢ The mutant residue is bigger than the wild-type residue.<br>➢ The residue is located on the surface of the protein; mutation of this residue can disturb interactions with other molecules or other parts of the protein.<br>➢ The torsion angles for this residue are unusual. Only glycine is flexible enough to make these torsion angles, mutation into another residue will force the local backbone into an incorrect conformation and will disturb the local structure.
rs1267664350	C220S	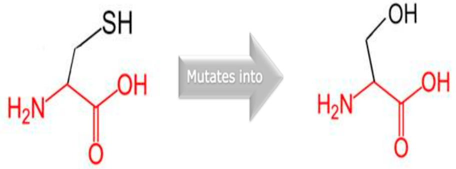	➢ The hydrophobicity of the wild-type and mutant residue differs.<br>➢ The mutation will cause loss of hydrophobic interactions in the core of the protein.

rs141153031	C233Y	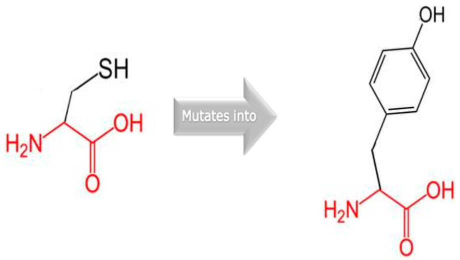	➢ The wild type and substituted amino acids differ in size.<br>➢ The mutant residue is bigger than the wild-type residue.<br>➢ The residue is located on the surface of the protein; mutation of this residue can disturb interactions with other molecules or other parts of the protein.<br>➢ The hydrophobicity of the wild type and mutant residue differs.<br>➢ The mutation might cause loss of hydrophobic interactions with other molecules on the surface of the protein.
rs1219119993	I240T	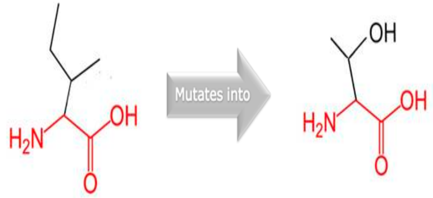	➢ The wild type and substituted amino acids differ in size.<br>➢ The mutant residue is smaller than the wild-type residue.<br>➢ The mutation will cause an empty space in the core of the protein.<br>➢ The hydrophobicity of the wild type and mutant residue differs.<br>➢ The mutation will cause loss of hydrophobic interactions in the core of the protein.
rs1458236572	E242G	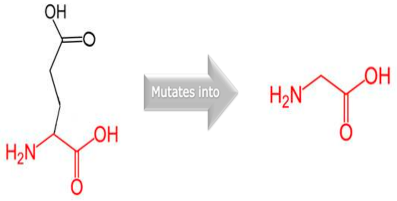	➢ The charge of the buried wild-type residue is lost by this mutation.<br>➢ The wild-type and substituted amino acids differ in size.<br>➢ The mutant residue is smaller than the wild-type residue.<br>➢ The mutation will cause an empty space in the core of the protein.<br>➢ The hydrophobicity of the wild type and mutant residue differs.<br>➢ The mutation will cause loss of hydrogen bonds in the core of the protein and as a result disturb correct folding.

Table S3. miRNA binding site prediction of non-coding SNPs in *Dectin-1*protein through PolymiRTS

Location	dbSNP ID	Variant type	Wobble base pair	Ancestral Allele	Allele	miR ID	Conservation	miRSite	Function Class	Exp Support	context+ score change
10269516	rs182562001	SNP	N	T							
					C	hsa-miR-4292	2	tattaCCAGGGAg	C	N	-0.172
						hsa-miR-4308	2	tattaCCAGGGAg	C	N	-0.182
						hsa-miR-6791-5p	2	tattaCCAGGGAg	C	N	-0.189
						hsa-miR-7113-3p	2	tattacCAGGGAG	C	N	-0.182
10269695	rs114659091	SNP	Y	A							
					G	hsa-miR-186-3p	6	tTTTGGGAtatgt	C	N	-0.103
						hsa-miR-4733-5p	2	ttTTGGGATAtgt	C	N	-0.365
10269719	rs185282370	SNP	N	T							
					C	hsa-miR-145-5p	2	acctcACTGGAAc	C	N	-0.125
						hsa-miR-1825	2	acctCACTGGAac	C	N	-0.136
						hsa-miR-199a-5p	2	acctCACTGGAac	C	N	-0.141
						hsa-miR-199b-5p	2	acctCACTGGAac	C	N	-0.138
						hsa-miR-5195-3p	2	acctcACTGGAAc	C	N	-0.128
10269746	rs58887007	SNP	N	A	A	hsa-miR-664a-3p	6	ctgatgATGAATA	D	N	-0.032
					T	hsa-miR-421	2	ctgaTGTTGAAta	C	N	-0.084
						hsa-miR-4272	2	ctgatGTTGAATA	C	N	-0.28
						hsa-miR-7159-5p	2	ctgaTGTTGAAta	C	N	-0.083
10269810	rs189798771	SNP	N	C	C	hsa-miR-2278	6	aaaCTGCTCTcat	D	N	-0.184
						hsa-miR-4712-3p	8	aaactgCTCTCAT	D	N	-0.164
					T	hsa-miR-146a-5p	2	aaactGTTCTCAt	C	N	-0.107
						hsa-miR-146b-5p	2	aaactGTTCTCAt	C	N	-0.107
						hsa-miR-4773	6	aaaCTGTTCTcat	C	N	-0.153
						hsa-miR-589-5p	7	aaactGTTCTCAt	C	N	-0.101
						hsa-miR-7153-5p	7	aaactGTTCTCAt	C	N	-0.09
10270149	rs187544967	SNP	N	T							
					A	hsa-miR-376a-5p	3	GAATCTAtttttt	C	N	-0.165
10270347	rs146370994	SNP	Y	A	A	hsa-miR-4277	2	ttatGAACTGAgg	D	N	-0.042

						hsa-miR-584-3p	2	ttatGAACTGAgg	D	N	-0.049
					G	hsa-miR-6736-3p	2	ttatGAGCTGAgg	C	N	-0.08
						hsa-miR-6787-3p	2	ttatgAGCTGAGg	C	N	-0.101
10270735	rs148551300	SNP	N	C	C	hsa-miR-1227-3p	2	GTGGCACAatctc	D	N	-0.394
						hsa-miR-5186	2	gtggcaCAATCTC	D	N	-0.053
						hsa-miR-550b-2-5p	2	gtGGCACAAtctc	D	N	-0.118
					T	hsa-miR-4633-5p	2	gtGGCATAAtctc	C	N	-0.111
10270773	rs10845048	SNP	N	G	G	hsa-miR-454-5p	2	gaGATAGGGtctc	D	N	-0.25
10270821	rs11053594	SNP	N	C	C	hsa-miR-146b-3p	6	tCAGGGCAgccaa	D	N	-0.182
						hsa-miR-18a-3p	7	tcAGGGCAGccaa	D	N	-0.196
						hsa-miR-3173-5p	6	tCAGGGCAgccaa	D	N	-0.178
						hsa-miR-6799-3p	6	tCAGGGCAgccaa	D	N	-0.189
						hsa-miR-7976	6	TCAGGGCAgccaa	D	N	-0.431
						hsa-miR-874-3p	6	tCAGGGCAgccaa	D	N	-0.235
					T	hsa-miR-10a-5p	6	tCAGGGTAgccaa	C	N	-0.163
						hsa-miR-10b-5p	6	tCAGGGTAgccaa	C	N	-0.16
10270822	rs11053595	SNP	N	G	G	hsa-miR-146b-3p	6	atCAGGGCAgcca	D	N	-0.182
						hsa-miR-18a-3p	7	atcAGGGCAGcca	D	N	-0.196
						hsa-miR-3173-5p	6	atCAGGGCAgcca	D	N	-0.178
						hsa-miR-6799-3p	6	atCAGGGCAgcca	D	N	-0.189
						hsa-miR-7976	6	aTCAGGGCAgcca	D	N	-0.431
						hsa-miR-874-3p	6	atCAGGGCAgcca	D	N	-0.235
					T	hsa-miR-3661	6	atCAGGTCAgcca	C	N	-0.147
						hsa-miR-631	6	atCAGGTCAgcca	C	N	-0.147
10270938	rs11053597	SNP	N	A	C	hsa-miR-4715-5p	3	cttgGCCAACTgt	C	N	-0.236
10270973	rs142132621	SNP	N	C	C	hsa-miR-192-5p	5	tgAGGTCAAgata	D	N	-0.174
						hsa-miR-215-5p	5	tgAGGTCAAgata	D	N	-0.174
						hsa-miR-6790-3p	4	tGAGGTCAagata	D	N	-0.185
						hsa-miR-6821-3p	4	tGAGGTCAagata	D	N	-0.177
					T	hsa-miR-3150a-5p	4	tGAGGTTAagata	C	N	-0.183
						hsa-miR-3150b-5p	4	tGAGGTTAagata	C	N	-0.183

Table S4. Functional enrichments analysis of *Dectin-1* network

Biology Process (Gene Ontology)

GO term	Description	count in network	strength	false discovery rate
GO:0001775	Cell activation	8 of 1075	1.12	3.39E-06
GO:0001817	Regulation of cytokine production	9 of 742	1.33	2.41E-08
GO:0001818	Negative regulation of cytokine production	3 of 280	1.28	0.0162
GO:0001819	Positive regulation of cytokine production	8 of 461	1.49	2.41E-08
GO:0001911	Negative regulation of leukocyte mediated cytotoxicity	2 of 20	2.25	0.0033
GO:0002092	Positive regulation of receptor internalization	2 of 27	2.12	0.0051
GO:0002218	Activation of innate immune response	5 of 142	1.8	2.90E-06
GO:0002223	Stimulatory C-type lectin receptor signaling pathway	4 of 111	1.81	5.53E-05
GO:0002237	Response to molecule of bacterial origin	7 of 330	1.58	8.08E-08
GO:0002250	Adaptive immune response	3 of 317	1.23	0.0215
GO:0002252	Immune effector process	8 of 969	1.17	1.74E-06
GO:0002253	Activation of immune response	6 of 385	1.44	6.67E-06
GO:0002274	Myeloid leukocyte activation	4 of 585	1.09	0.0088
GO:0002312	B cell activation involved in immune response	3 of 45	2.07	0.00022
GO:0002316	Follicular B cell differentiation	2 of 2	3.25	0.00019
GO:0009620	Response to fungus	4 of 61	2.07	8.87E-06
GO:0002366	Leukocyte activation involved in immune response	6 of 626	1.23	6.57E-05
GO:0002376	Immune system process	10 of 2481	0.86	3.04E-06
GO:0002429	Immune response-activating cell surface receptor signaling pathway	5 of 311	1.46	6.61E-05
GO:0002520	Immune system development	6 of 652	1.21	7.71E-05
GO:0002521	Leukocyte differentiation	5 of 338	1.42	8.73E-05
GO:0002532	Production of molecular mediator involved in inflammatory response	2 of 16	2.35	0.0023
GO:0002683	Negative regulation of immune system process	4 of 450	1.2	0.0039

GO:0002684	Positive regulation of immune system process	9 of 949	1.23	6.06E-08
**Molecular function (Gene Ontology)**				
GO:0001540	Amyloid-beta binding	3 of 76	1.85	0.011
GO:0019865	Immunoglobulin binding	2 of 23	2.19	0.0277
GO:0001530	Lipopolysaccharide binding	2 of 34	2.02	0.0483
GO:0001875	Lipopolysaccharide immune receptor activity	2 of 5	2.85	0.0097
GO:0050135	NAD(P)+ nucleosidase activity	2 of 16	2.35	0.0236
GO:0061809	NAD+ nucleotidase, cyclic ADP-ribose generating	2 of 16	2.35	0.0236
GO:0070492	Oligosaccharide binding	2 of 16	2.35	0.0236
GO:0038187	Pattern recognition receptor activity	3 of 24	2.35	0.0012
GO:0051219	Phosphoprotein binding	3 of 87	1.79	0.0122
GO:0005102	Signaling receptor binding	6 of 1581	0.83	0.0277
GO:0035325	Toll-like receptor binding	2 of 12	2.47	0.0168
**Cellular Component (Gene Ontology)**				
GO:0009986	Cell surface	5 of 824	1.03	0.031
GO:0005886	Plasma membrane	10 of 5314	0.52	0.031

KEGG pathways

**Pathways**	**Description**	**count in network**	**strength**	**false discovery rate**
hsa04662	B cell receptor signaling pathway	3 of 78	1.84	0.00074

hsa04625	C-type lectin receptor signaling pathway	5 of 102	1.94	3.36E-07
hsa04666	Fc gamma R-mediated phagocytosis	3 of 90	1.77	0.00093
hsa05170	Human immunodeficiency virus 1 infection	3 of 204	1.42	0.004
hsa04750	Inflammatory mediator regulation of TRP channels	2 of 94	1.58	0.0147
hsa04650	Natural killer cell mediated cytotoxicity	2 of 121	1.47	0.0194
hsa04064	NF-kappa B signaling pathway	3 of 101	1.72	0.0011
hsa04151	PI3K-Akt signaling pathway	3 of 350	1.18	0.0121
hsa04620	Toll-like receptor signaling pathway	2 of 101	1.55	0.0157

Disease - genes associations

Diseases	Description	count in network	strength	false discovery rate
DOID:0050117	Disease by infectious agent	4 of 317	1.35	0.0344
DOID:0050292	Primary systemic mycosis	2 of 9	2.6	0.0344
DOID:2272	Vulvovaginal candidiasis	2 of 2	3.25	0.0075
